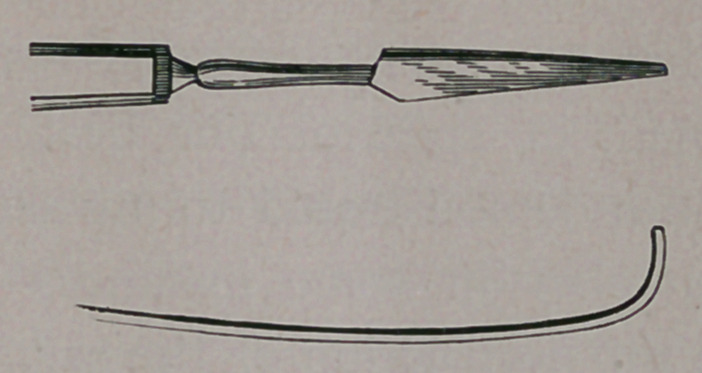# Treatment of Strictures of the Nasal Duct

**Published:** 1880-09

**Authors:** Lucien Howe


BUFFALO

MEDICAL AND SURGICAL JOURNAL.

VOL. XX.—SEPTEMBER, 1880.—NO. 2.

### Original Communications.

### TREATMENT OF STRICTURES OF THE NASAL DUCT.

BY LUCIEN HOWE, M. D.

IT is often the case that when a disease is difficult to treat successfully, the text books enumerate a proportionately large number of methods and medicines proposed for its cure.

Thus do we find it with the diseases of the lachrymal apparatus. The ease of diagnosis in this class of troubles brings them clearly and frequently before the practitioner. The "watery eye" in the simple form, or the mucous discharge which can easily be pressed out into the inner angle of the eye, or the inflammatory symptoms which accompany the retention of such secretion; all these, alone or combined, point too clearly to some obstruction of the tear passage. There is no doubt as to the cause of the symptoms, but the question as to how the difficulty should be treated, has been variously answered.

This field of inquiry has been so thoroughly investigated by surgeons of eminence, from the days of Scarpa to the present time, that it is almost impossible to call attention to any point which has thus far escaped notice. Too many chapters and too

4

many books have already been written, covering the entire ground, for one to review even superficially, the different methods proposed, in a single paper of moderate length.

There are many questions concerning the most appropriate treatment for this class of cases, in their earlier stages, which it might be interesting to consider, as to the disadvantages of Bowman's probes as to the benefits derived from a tentative plan, and as to the value of other methods usually employed for the treatment of the more simple forms. Concerning these, however, I would only observe in passing, that the tendency at present, is toward a rather more conservative method than that taught and practiced in general only a few years ago. Recent writers, like Carter* and Wecker,† simply express the best judgment of the profession in counseling a certain caution in dealing with such cases.

It may be well to follow Becker's plan, of simply passing a sound through the upper canaliculus without previously dividing it; it is often still better to enlarge the lower punctum, or slit up that canaliculus only so far, and in such a direction as to allow the tears more readily to enter this part of the canal; but that is all, I think, we are warranted in doing, in an operative way, for the more simple forms of the disease. The practice of indiscriminate cutting, in every case presented, and then forcing a probe along the delicate lining of mucous membrane, is fortunately becoming less frequent.

But when, from long neglect or other cause, the more acute symptoms of a dacryosystitis are well developed, and especially when these show a tendency to frequent recurrence, then some more decided measures are demanded, And it is to the treatment of this condition that I would particularly refer.

For the favorable result, in several cases of the most pronounced type, have led me to think that some variety of Stilling's method should always be tried; that with a proper knife

* Carter on the Eye. Green, p. 202.

† Chirurgie Oculaire. Wecker, p. 395.

the procedure is easily executed, and with persistent use of probes the proportion of good results is increased. To these points, therefore, I would briefly call attention.

For in the more aggravated forms of stricture of the nasal duct, we are left with a disagreeable alternative.

On the one hand, we must completely destroy the sac by caustic (*Beer Magne Agnew*) or extirpate the lachrymal gland (*Lawson*); or, on the other hand, an effort must be made to remove the obstruction, although such attempts are always tedious, usually painful and in the majority of cases, unsuccessful. The methods first mentioned, although frequently recommended in the text books, are apt to appear so severe, that the patient instinctively shrinks from such treatment, and it is not surprising that the ingenuity of surgeons should be taxed to find some means of otherwise overcoming the difficulty. The only plan which offers any prospect of relief is undoubtedly some form of the one first proposed by Stilling*, and known otherwise as *stricturotomy*. It occurred to him that strictures in the nasal duct could be divided internally in the same manner as those in the urethra. Accordingly, after slitting up the canaliculus he thrust a narrow knife down through the sac into the duct, thus cutting the stricture, and then, turning the edge in different directions, divided the obstruction freely.

The accumulating secretion was then pressed into the nostril sufficiently often to allow the walls of the sac to regain their former tonicity and thus contracting spontaneously, to expel the abnormal collection.

It is by no means certain that the "obstruction" can, in a strict pathological sense, be considered a stricture, and it is a question, as to whether the large opening into the upper part of the sac, and not the division of this "obstruction," is really the cause of improvement. But such considerations have little practical importance in this connection. The real fact is, that

* Heilung der Verengerung der Thraenenwege.

in a certain number of cases, this treatment gives relief, when we would otherwise have to resort to much harsher means.

The objection to Stilling's plan, however, is the very important one, that it is often entirely useless. I am inclined to think the simple division of the stricture and subsequent pressure, is followed by a return of the difficulty in at least 75 per cent. of the cases. With a view to facilitate the introduction of the knife, several modifications of its form have been proposed, and with the hope of increasing the proportion of fortunate results, various changes have been made by different surgeons in the details of the method.

Nearly every one adopts for himself some particular method and is inclined to hold tenaciously to it. It has occurred to me, however, during the past two years to treat at least eight cases in which the symptoms were so aggravated as to warrant the most radical measures in the event of the stricture not yielding to treatment, and the almost uniformly good results seemed to warrant attention being called to one or two details.

In the first place I made it a point, after dividing the stricture, internally, to introduce at once a rather large probe, number five or six. There is nothing original in this, and if I mistake not, it has long been practiced at the New York Eye and Ear Infirmary and elsewhere. But I early came to the conclusion that the details of this apparently simple manoeuvre was a matter of much importance. Under these circumstances, the mucous membrane of the sac is swollen, often thickened or even granular. Slight projections into the sac, or diverticula in different directions are not uncommon. With such a condition it is exceedingly easy to scrape off comparatively large portions of the mucous lining, to tear it away from the bony walls, or to make false passages in different directions, any of which accidents would result in much greater injury to its structure than the clean, smooth cut.

Indeed, after withdrawing the knife, I have several times found it almost impossible to guide the probe through the opening already made.

With the view to lessen this difficulty, I have caused a deep groove to be cut along the narrow back of the blade. (See figure.)

Then, after passing the knife well down through the stricture it is allowed to remain there while the point of the probe is applied to the back of the blade, and this now acting as a grooved director brings the point directly to the opening.

Frequently when the walls are narrowed by irregularity of the bony canal,* or even by unusual thickening of the mucous membrane, it is necessary to gradually withdraw the knife as the probe is advanced, but this, of course, can be readily done.

Moreover, after the probe is once in place, I prefer to leave it for three or four days. If it is so short as to protrude only a little distance beyond the internal angle, or especially, if the upper end has previously been bent at an angle (see figure), it will hardly be noticeable, and will not prevent frequent pressure being made on the sac to evacuate its contents. If its presence proves irritating, it can easily be removed, and when an attempt is made to introduce a still larger size, this can usually be accomplished with greater ease than at first. As the secretion gradually lessens and the canal resumes its more normal condition, the use of the probe can be discontinued, or if introduced at all, may be removed after a few moments.

It would be difficult to say how the mere presence of a probe in the canal, for a few days or weeks, can produce absorption of the thickened membrane (if indeed it does so), so as to hinder that contraction which in the end is almost sure to occur; or on the other hand to explain exactly why the probe itself does not partially obstruct the opening made in the stricture, and thus

* Graefe und Saemisch Handbuch der Ophthalmologie, Vol. I, p. 100, and Vol. III, p. 490.

impede the evacuation of the sac. Such theoretical objections have occurred to me as worthy of some consideration, and it is quite probable that further study of these or similar points will tend to increase that percentage of good results, which is unfortunately too small, in spite of our best efforts. But the practical fact has been demonstrated to my satisfaction that this general plan gives relief in some instances where we would otherwise resort to the destruction of the sac, or removal of the gland. It seemed to me, therefore, that an attempt at some form of Stilling's method should always be made before resorting to destruction of the sac, that the grooved knife is more convenient than the other forms, and that the continued presence of the probe, for at least a short time, does tend to keep the canal permanently open.

## Figures and Tables

**Figure f1:**